# Percutaneous Cryoablation in the Liver: A Meta-Analysis and Review of Safety with a Focus on Incidence of Cryoshock and Major Complications

**DOI:** 10.1007/s00270-024-03869-9

**Published:** 2024-10-15

**Authors:** Johannes Kolck, Daniel Schulze, Michael Brönnimann, Matthias Fürstner, Uli Fehrenbach, Federico Collettini, Bernhard Gebauer, Timo A. Auer

**Affiliations:** 1 Department of Radiology, Charité-Universitätsmedizin Berlin, Charité Campus Virchow (CVK), Augustenburger Platz 1, 13353 Berlin, Germany; 2 Institute of Biometry and Clinical Epidemiology, Charité-Universitätsmedizin Berlin, Berlin, Germany; 3 Department of Diagnostic, Interventional and Paediatric Radiology, Inselspital, Bern University Hospital, University of Bern, 3010 Bern, Switzerland; 4 Department of Radiology, Klinikum Klagenfurt, Klagenfurt am Wörthersee, Austria; 5 Berlin Institute of Health at Charité-Universitätsmedizin Berlin, Berlin, Germany

**Keywords:** Liver cryoablation, Cryoshock, Local treatment, Liver malignancies

## Abstract

**Purpose:**

The aim of the present meta-analysis was to systematically determine the overall complication rate and incidence of cryoshock in patients undergoing cryoablation of the liver.

**Methods:**

A systematic review and meta-analysis adhering to the PRISMA guidelines and focusing on studies of cryotherapy for liver malignancies published after 2000 were conducted. PubMed, Web of Science, Embase, and Scopus were systematically searched for articles reporting incidences of adverse events associated with percutaneous cryoablation in patients with liver malignancies. Data extraction and screening were independently conducted by two reviewers, who resolved discrepancies through consensus. Statistical analysis was performed to assess heterogeneity and pooled complication rates and included a moderator analysis to explore factors influencing the occurrence of complications.

**Results:**

The initial search yielded 4,145 articles, of which 26 met our inclusion criteria. From these 26 articles, pooled data on 4,029 patients were extracted. Variance between studies reporting cryoshock was low (I^2^ = 13.15%), while variance among studies reporting major complications was high (I^2^ = 82.52%). The pooled weighted proportion of major complications was 4.71% while that of cryoshock was as low as 0.265%. Moderator analysis identified publication year as the only moderator for major complications and no moderator for the occurrence of cryoshock.

**Conclusion:**

Analysis of currently available evidence indicates that cryoablation has a relative safe profile with a pooled incidence of major complications below 5%. Cryoshock occurred in less than 0.3% of procedures and was not reported for liver lesions smaller than 3 cm.

## Introduction

Locoregional ablative tumor therapies are increasingly important because they are minimally invasive and offer a curative approach for patients ineligible for surgery [1]. Techniques used in the liver include radiofrequency ablation (RFA), microwave ablation (MWA), irreversible electroporation (IRE), and cryoablation. RFA and MWA are well established as first-line treatments for certain malignancies, including hepatocellular carcinoma [2]. Cryoablation, unlike traditional hyperthermic techniques, employs hypothermia to induce cell death through both direct thermal damage and indirect mechanisms that alter the cellular microenvironment [3]. In addition, cryoablation provides superior intraprocedural visualization and causes less pronounced direct thermal injury at the periphery of the ablation zone, improving its safety profile. These features make cryoablation particularly suitable for treating lesions close to the bile ducts or intestines, where the use of hyperthermic procedures could pose a higher risk [1, 4]. Currently, cryoablation is experiencing a renaissance, especially in the treatment of renal cell carcinoma. Yet, it is used cautiously in patients with liver lesions [1, 2, 5]. This hesitancy is mainly due to the risk of cryoshock, a potentially fatal tumor lysis syndrome specifically associated with cryoablation in the liver [6]. The precise pathophysiological mechanisms underlying cryoshock remain unclear, posing challenges for clinicians in anticipating and managing its occurrence during liver procedures [7, 8] (Tables 1 and 2).

**Table 1:** Characteristics of study populations investigated in the 26 studies included in our analysis

ID	Author and year	Study type	Imaging modality	Sample size	Female	Male	No of lesion
1	Adam, R. 2002	Retrospective	US	31	11	20	42
2	Chen, H. 2011	Retrospective	US	66	11	55	152
3	Cui, W. 2018	Retrospective	CT	56	7	49	–
4	Dunne, R. 2014	Retrospective	CT/MRI	25	8	17	39
5	Ei, S. 2015	Prospective	US	55	17	38	–
6	Glazer, D. 2017	Retrospective	CT, MRI	186	95	91	299
7	Kim, G. 2016	Retrospective	US	28	3	25	–
8	Kim, R. 2018	Retrospective	US/CT	58	10	48	48
9	Ko, S. 2020	Retrospective	US	25	4	21	25
10	Li, Z. 2013	Retrospective	CT/US	24	8	16	-
11	Littrup, P. 2016	Prospective	CT	212	116	96	370
12	Ma, J. 219	Retrospective	CT/US	66	17	49	69
13	Mala, T. 2001	Prospective	MRI	6	–	–	12
14	Pusceddu, C. 2022	Prospective	CT	49	26	23	54
15	Qi, C. 2020	Retrospective	CT	73	13	60	73
16	Rong, G. 2015	Retrospective	CT/US	1595	278	1317	2123
17	Silverman, S. 2004	Retrospective	MRI	9	5	4	9
18	Wang, C. 2014	Prospective	CT	180	40	140	228
19	Wang, L. 2019	Prospective	MRI	37	11	26	37
20	Wei, J.2020	Retrospective	CT	60	6	54	–
21	Xu, K. 2003	Retrospective	US	65	18	47	105
22	Xu, K. 2008	Prospective	CT/US	326	83	243	–
23	Xu, K. 2009	Retrospective	US	420	110	310	–
24	Yang, Y. 2012	Retrospective	US	300	44	256	408
25	Zeng, J. 2018	Prospective	CT/US	60	8	52	–
26	Zhang, W. 2014	Retrospective	CT	17	17	0	39
Total (n)				4029	966	3057	4132
Total (%)					24.0%	75.9%	

ID	Proportion of HCC	Mean lesion size (mm)	Minor complications	Minor complications (%)	Major complications	Major complications (%)	Cryoshock	Relevant bleeding	Death
1	18	2.22	5	16.1	5	16.1	0	2	1
2	66	2.80	6	9.1	2	3.0	0	1	0
3	56	13.40	79	141.1	1	1.8	0	1	0
4	25	2.80	11	44.0	6	24.0	0	0	0
5	55	2.50	0	0.0	–	–	0	0	0
6	50	2.50	80	43.0	31	16.7	0	13	1
7	28	1.74	5	17.9	1	3.6	0	1	0
8	58	1.30	4	6.9	–	–	0	0	0
9	25	1.50	9	36.0	–	–	0	0	0
10	24	2.50	–	–	–	–	0	0	0
11	36	2.80	17	8.0	10	4.7	3	3	4
12	15	2.10	19	28.8	1	1.5	0	0	0
13	0	5.00	7	116.7	2	33.3	0	0	0
14	14	2.15	7	14.3	0	0.0	0	0	0
15	33	2.60	32	43.8	6	8.2	0	0	0
16	1595	3.80	380	23.8	94	5.9	0	9	2
17	0	3.01	–	–	–	–	0	0	0
18	180	–	239	132.8	5	2.8	0	2	0
19	37	2.90	102	275.7	3	8.1	0	1	0
20	60	11.80	79	131.7	1	1.7	0	0	0
21	65	7.30	6	9.2	3	4.6	0	1	0
22	0	–	7	2.1	20	6.1	1	5	7
23	420	4.60	39	9.3	57	13.6	0	16	0
24	300	6.47	6	2.0	21	7.0	6	5	2
25	60	6.83	93	155.0	–	–	0	0	0
26	0	3.50	25	147.1	–	–	0	0	0
Total (n)	3220		1257		269		10	60	17
Total (%)	79.9%		33.9%		6.7%		0.2%	1.5%	0.4%

**Table 2:** Summary of data on complications reported in the studies included in our meta-analysis

ID	Author and Year	Study type	Imaging modality	Sample size	Female	Male	No of lesion	Proportion of HCC
1	Adam, R. 2002	Retrospective	US	31	11	20	42	18
2	Chen, H. 2011	Retrospective	US	66	11	55	152	66
3	Cui, W. 2018	Retrospective	CT	56	7	49	–	56
4	Dunne, R. 2014	Retrospective	CT/MRI	25	8	17	39	25
5	Ei, S. 2015	Prospective	US	55	17	38	–	55
6	Glazer, D. 2017	Retrospective	CT, MRI	186	95	91	299	50
7	Kim, G. 2016	Retrospective	US	28	3	25	–	28
8	Kim, R. 2018	Retrospective	US/CT	58	10	48	48	58
9	Ko, S. 2020	Retrospective	US	25	4	21	25	25
10	Li, Z. 2013	Retrospective	CT/US	24	8	16	–	24
11	Littrup, P. 2016	Prospective	CT	212	116	96	370	36
12	Ma, J. 219	Retrospective	CT/US	66	17	49	69	15
13	Mala, T. 2001	Prospective	MRI	6	–	–	12	0
14	Pusceddu, C. 2022	Prospective	CT	49	26	23	54	14
15	Qi, C. 2020	Retrospective	CT	73	13	60	73	33
16	Rong, G. 2015	Retrospective	CT/US	1595	278	1317	2123	1595
17	Silverman, S. 2004	Retrospective	MRI	9	5	4	9	0
18	Wang, C. 2014	Prospective	CT	180	40	140	228	180
19	Wang, L. 2019	Prospective	MRI	37	11	26	37	37
20	Wei, J.2020	Retrospective	CT	60	6	54	–	60
21	Xu, K. 2003	Retrospective	US	65	18	47	105	65
22	Xu, K. 2008	Prospective	CT/US	326	83	243	–	0
23	Xu, K. 2009	Retrospective	US	420	110	310	–	420
24	Yang, Y. 2012	Retrospective	US	300	44	256	408	300
25	Zeng, J. 2018	Prospective	CT/US	60	8	52	–	60
26	Zhang, W. 2014	Retrospective	CT	17	17	0	39	0
Total (n)				4029	966	3057	4132	3220
Total (%)					24.0%	75.9%		79.9%

ID	Mean lesion size (mm)	Minor complications	Minor complications (%)	Major complications	Major complications (%)	Cryoshock	Relevant bleeding	Death
1	2.22	5	16.1	5	16.1	0	2	1
2	2.80	6	9.1	2	3.0	0	1	0
3	13.40	79	141.1	1	1.8	0	1	0
4	2.80	11	44.0	6	24.0	0	0	0
5	2.50	0	0.0	–	–	0	0	0
6	2.50	80	43.0	31	16.7	0	13	1
7	1.74	5	17.9	1	3.6	0	1	0
8	1.30	4	6.9	–	–	0	0	0
9	1.50	9	36.0	–	–	0	0	0
10	2.50	–	–	–	–	0	0	0
11	2.80	17	8.0	10	4.7	3	3	4
12	2.10	19	28.8	1	1.5	0	0	0
13	5.00	7	116.7	2	33.3	0	0	0
14	2.15	7	14.3	0	0.0	0	0	0
15	2.60	32	43.8	6	8.2	0	0	0
16	3.80	380	23.8	94	5.9	0	9	2
17	3.01	–	–	–	–	0	0	0
18	–	239	132.8	5	2.8	0	2	0
19	2.90	102	275.7	3	8.1	0	1	0
20	11.80	79	131.7	1	1.7	0	0	0
21	7.30	6	9.2	3	4.6	0	1	0
22	–	7	2.1	20	6.1	1	5	7
23	4.60	39	9.3	57	13.6	0	16	0
24	6.47	6	2.0	21	7.0	6	5	2
25	6.83	93	155.0	–	–	0	0	0
26	3.50	25	147.1	–	–	0	0	0
Total (n)		1257		269		10	60	17
Total (%)		33.9%		6.7%		0.2%	1.5%	0.4%

Major complications are shaded in gray

DIC = Disseminated intravascular coagulation. PLE = Pleural effusion. PV = Portal vein

The aim of this meta-analysis was to assess the safety of liver cryoablation by systematically investigating overall complication rates and the incidence of cryoshock. Additionally, the study aimed at providing a comprehensive review of published data.

## Materials and Methods

### Literature Search and Data Acquisition

**Fig. 1:**
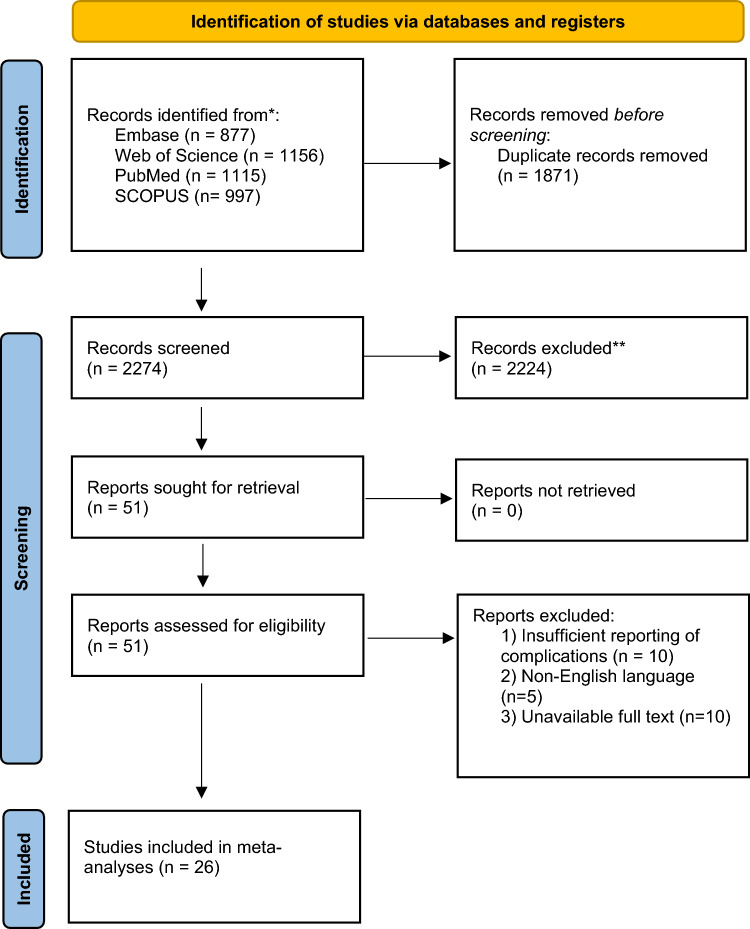
Flow chart showing selection process for inclusion of articles in meta-analysis

This systematic review and meta-analysis followed the PRISMA standards [9]. A comprehensive literature search for studies on cryotherapy for liver malignancies published after 2000 was conducted in PubMed, Web of Science, Embase, and Scopus. Search terms included variations of “cryotherapy,” “cryoablation,” “liver,” “hepatic metastasis,” “hepatocellular carcinoma,” and “cholangiocarcinoma.” Studies included were randomized controlled trials (RCTs), case–control studies, cohort studies, and cross-sectional studies investigating complications of percutaneous cryoablation in adults (aged 18 or older). Exclusion criteria were reviews, case reports, abstracts, conference papers, studies with fewer than five patients, and animal studies. Titles and abstracts were screened, followed by full-text reviews. Studies comparing cryoablation with other modalities were included if they reported on cryoablation. Two reviewers (TAA and JK) independently performed screening and data extraction, resolving discrepancies by consensus. The search strategy and selection process are summarized in a PRISMA flowchart (Fig. 1). Extracted data included study characteristics, participant demographics, intervention details, and complications.

### Classification of Complications

The systematic literature review revealed that very few of the included studies employed standardized classification systems for adverse events; and in most studies, such systems were not used at all. Most investigators merely described the occurrence and immediate consequences of adverse events, providing little information on long-term outcomes. To address this inconsistency and enhance the rigor of the current analysis, the investigators agreed to use the modified Society of Interventional Radiology (SIR) classification system for adverse events [10, 11]. In accordance with this classification system, reported events were subdivided into minor (SIR: mild to moderate) and major (SIR: severe, life-threatening/disabling and patient death) complications. Accordingly, cyoreaction and cryoshock were classified as minor and major complications, respectively.

### Risk-of-*Bias* Assessment and Statistical Analysis

Heterogeneity among studies was assessed using the Cochrane Q test. The I^2^ statistic was utilized to quantify between-study variation or heterogeneity. A restricted maximum-likelihood model was used to calculate the pooled weighted proportions of complications and cryoshock; they are reported with their 95% confidence interval (CI). A moderator analysis was conducted to explore how various factors such as gender distribution, entity of ablated liver tumor, lesion size, application of cryoablation in combination with transarterial chemoembolization (TACE), guidance devices, ablation at special sites, and publication year may have affected the reported outcomes of cryoablation in liver tumor management. This analysis was performed to identify potential sources of heterogeneity or variation in treatment effectiveness across different studies and patient populations. Results of the meta-analysis are presented as forest plots. A risk of bias assessment was conducted using the ROB-2 tool [12], considering the following give domains: (1) bias from incomplete description of the study population; (2) bias from incomplete data on the ablation procedure; (3) bias from incomplete data on ablated lesions (size and entity); (4) bias from failure to use a standardized reporting system for adverse events; and (5) bias from incomplete data on major complications. Publication bias was assessed using funnel plots. As conventional funnel plots are considered inaccurate for meta-analyses of proportion studies with low proportion outcomes, funnel plots of study size against log odds were calculated [13]. All analyses were performed using Jamovi version 2.3 (The jamovi project, Sydney, Australia).

## Results

### Included Studies and Risk-of-*Bias* Assessment

**Fig. 2:**
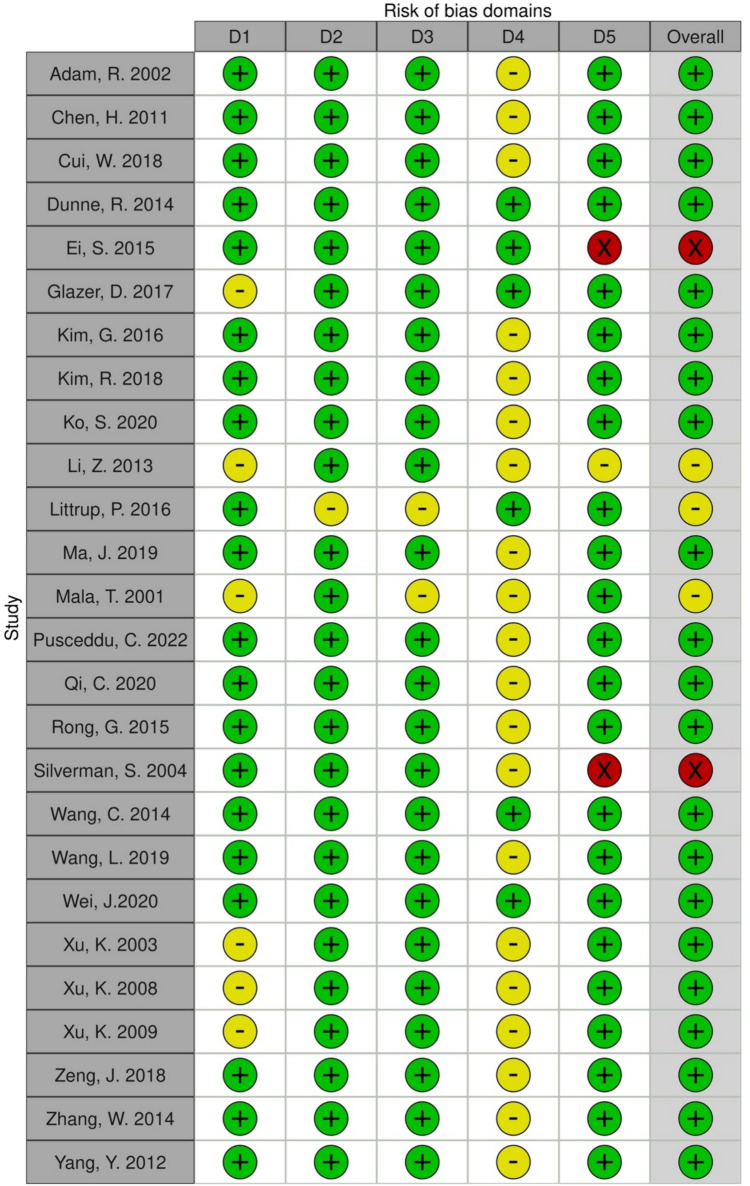
Graph displaying domain-level judgments for each specific outcome, indicating bias in the reporting of cohort/patient (D1) and intervention details (D2), lesion characteristics (D3), and reporting of major complications (D5), as well as the utilization of standardized standard reporting system for complications (D4)

**Fig. 3:**
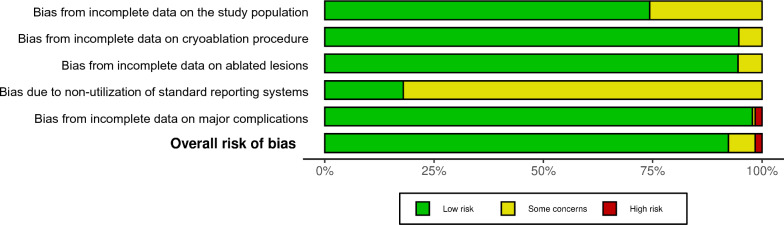
Weighted bar plots illustrating the distribution of risk-of-bias judgments within each bias domain

Aggregated data on a total of 4029 patients reported in 26 distinct articles that were selected as described above (Fig. 1) were included in our meta-analysis. The articles included presented eight prospective and eighteen retrospective studies [4, 14–38]. Overall, there was a low risk of bias according to the ROB-2 tool. Most of the studies included exhibited a low risk of bias regarding the reporting of cohort/patient (D1) and intervention details (D2), lesion characteristics (D3), and the reporting of major complications (D5). However, raters expressed some concern regarding most studies because investigators reported complications in detail but did not adhere to a standard reporting system (D4). Additionally, two studies did not report any major complications at all. The domain-level judgments are depicted in Fig. 2. Figure 3 presents the weighted bar plots of the distribution of risk-of-bias judgements within each bias domain.

### Patient Data

The number of patients per study ranged from 6 to 1595 patients. A total of 966 women and 3057 men were included in this analysis. Gender distribution remained unclear for six patients, as Mala et al. did not provide this information [25]. Mean patient age in the 26 studies ranged from 29 to 88 years. The average diameter of treated lesions, reported for 3532 patients, ranged from 1.3 to 13.4 cm. Average lesion diameter was not reported for 506 patients (studies of Xu et al. and Wang et al. [30, 33]. Instead, Xu et al. reported 107 patients with diameters exceeding 5 cm, 124 patients with diameters of 3–5 cm, and 95 individuals with lesion diameters < 3 cm. Wang et al. treated 34 patients with lesions below 2 cm and 146 individuals with lesions between 2 and 4 cm in diameter. In our analysis, hepatocellular carcinoma, in a total of 2920 patients, was the most commonly treated liver malignancy, followed by metastases from colorectal, esophageal, stomach, and breast cancer.

### Cryoablation Technique

Percutaneous cryoablation procedures were performed employing various cryo-devices. The CRYO-HIT device (Galil Medical Ltd, Yokneam, Israel) was employed in 13 studies, followed by Cryocare Operative System (Endocare, CA, USA), which was used in 10 studies, while the IceRod PLUS (Boston Scientific International, Netherlands) and AT-2008-II devices (AccuTarget MediPharma, Shanghai, China) featured in only one study each. Littrup et al. did not specify the utilized cryo-system [23].Guidance during cryoablation procedures primarily relied on computed tomography (CT) and ultrasound (US), less frequently on magnetic resonance imaging (MRI). Cryoprobes with diameters ranging from 1.47 to 8 mm were used. Some authors reported sealing of puncture tracts with fibrin glue, hemostatic gel, surgical or gelfoam after withdrawal of cryoprobes [22, 28, 30, 34, 35, 37].

### Complications

**Fig. 4:**
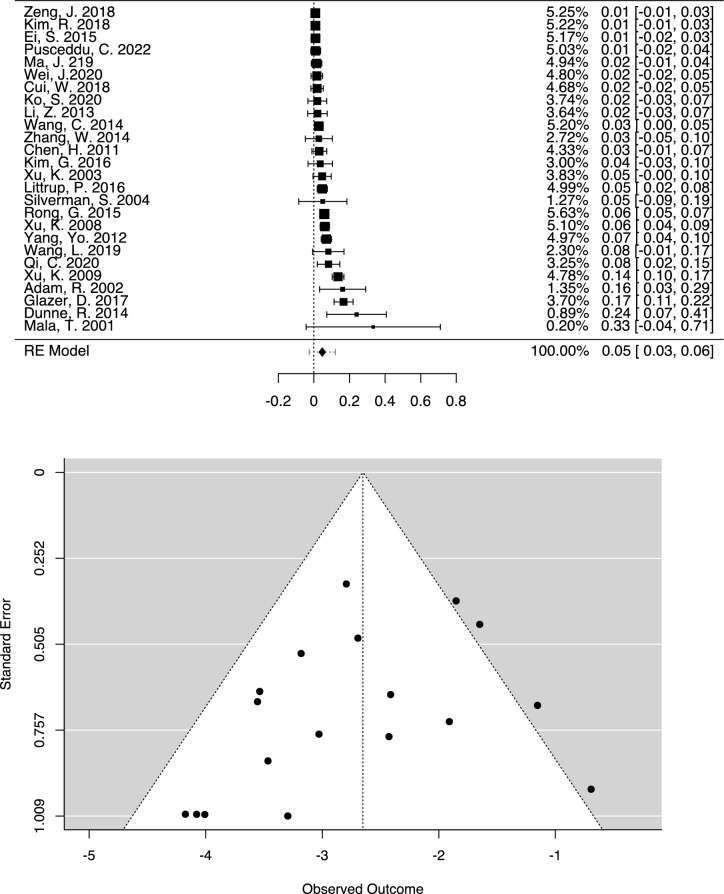
Proportional forest plot illustrating the incidence of major complications during or after hepatic cryoablation. The overall incidence of major complications was low at 4.71%, but showed considerable variability. The funnel plot showed a symmetrical distribution of publications. (Re Model: Random effect)

In the 4029 patients who underwent cryoablation, a total of 1275 incidences of minor complications, 269 incidences of major complications, which included 10 instances of cryoshock, and 17 deaths attributed to the procedure were reported. The pooled weighted proportions were 4.71% for all major complications (Fig. 4), 1.39% for bleedings requiring interventional management, and 0.28% for deaths. In the moderator analysis, the year of publication was identified as the only significant factor affecting the occurrence of major complications (*p* = 0.015; Table 3). Papers published more recently reported fewer major complications. Detailed data on the broad variety of complications reported in the studies analyzed are summarized in Table 2. Heterogeneity among studies reporting complications was high (I^2^ = 82.87%; Q = 118.752; *p* < 0.001). Egger’s test was not statistically significant (*P* = 0.131), suggesting an absence of publication bias. This was further supported by the symmetrical funnel plot.

**Table 3:** Summary of moderator analysis for major complications

Moderator	Estimate	Se	Z	p
Female gender	1.45E-04	1.47E-04	0.988	0.323
Entity	− 0.00441	0.026	-0.17	0.865
Lesion size	0.00272	0.00971	0.28	0.78
Guidance device	0.0259	0.0185	1.404	0.16
Combination w. TACE	2.16E-06	2.27E-05	0.0952	0.924
Ablation at special site	− 0.0738	0.0438	− 1.69	0.092
Publication year	− 0.00636	0.00261	− 2.44	0.015

The publication year was the only significant moderator (*p* = 0.015). All other listed moderators had no statistically significant influence

### Cryoshock

**Fig. 5:**
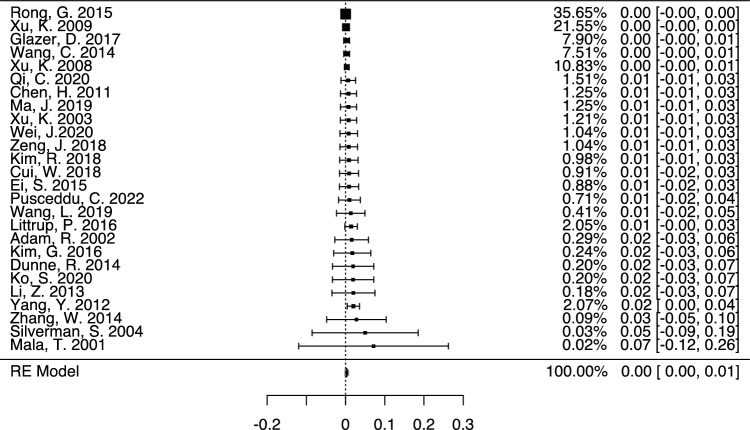
Proportional forest plot depicting the incidence of cryoshock complications during or following hepatic cryoablation. The overall occurrence of serious complications was minimal, at less than 0.3%. (Re Model: Random effect)

Three studies included in our meta-analysis reported a combined total of 10 patients who experienced Cryoshock, and four of the patients died [23, 33, 39], corresponding to a high fatality rate of 40%. The pooled weighted proportion of cryoshock over all studies was 0.265% (Fig. 5). Heterogeneity among studies reporting cryoshock was low (I^2^ = 13.15%), and no significant moderators were identified. Egger’s test did not detect statistical significance (*P* = 0.455), indicating the lack of publication bias. Littrup et al. reported three incidences: an 81-year-old cirrhotic patient who died of uncontrolled hemorrhage immediately following cryoablation of a large hepatoma measuring 6.3 cm and two deaths occurring 22 and 10 days after cryoablation. One of these patients had a single tumor of 3.4 cm and a hemoglobin level of 11.8 dL/g with a platelet count of 77,000 while the other patient had three lesions with diameters of 4.3, 3.7, and 1.9 cm, corresponding to a total tumor diameter of 9.9 cm [23]. Xu et al. reported a lethal Cryoshock in a patient with eight large lesions, without providing further details [33]. Yang et al. described more typical symptoms of Cryoshock in six patients, who had a tumor area of 50–60 cm^2^ (representing estimated diameters of 7.98–8.74 cm) and developed chills, fever, tachycardia, tachypnea, and temporary renal damage. All six patients were covered with an electric blanket and received oxygen inhalation and recovered following intravenous administration of atropine and 5% sodium bicarbonate [39].

## Discussion

While cryoablation is gaining traction, particularly for treating renal cell carcinoma, it is still used cautiously in the liver due to the risk of cryoshock, a dreaded tumor lysis syndrome with life-threatening consequences. Our meta-analysis investigated the overall complication rate and incidence of cryoshock in liver cryoablation in 26 studies with 4,029 patients. There were a total of 269 major complications, including 60 instances of bleeding requiring intervention, 10 cases of cryoshock, and 17 fatalities. The pooled weighted proportions were 4.71% for all major complications, 1.39% for bleedings requiring interventional management,and 0.28% for deaths. The pooled incidence of cryoshock was 0.27%, and all cases occurred in patients with lesions exceeding 3 cm in diameter. No significant moderators were identified for cryoshock, whereas publication year emerged as a significant moderator for major complications (*p* = 0.041), indicating improved safety outcomes over time, likely due to the accumulation of experience and refinement of cryoablation techniques.

**Fig. 6:**
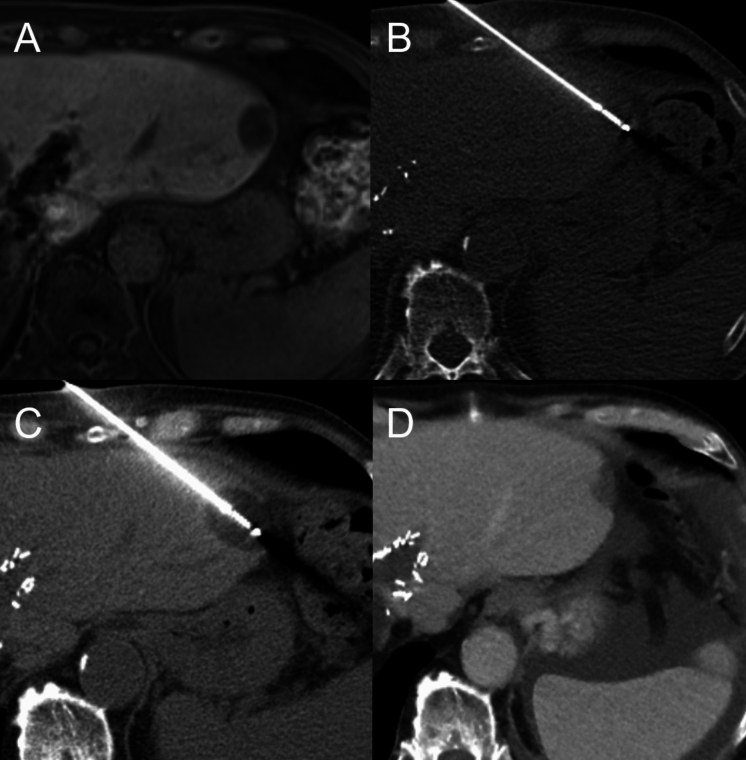
72-year-old male patient with recurrence of a colorectal liver metastasis in the left liver lobe. **A** Preinterventional axial MRI (hepatobiliary phase) shows a single recurrent metastasis in the left liver lobe located directly adjacent to the left colon. **B** Intraprocedural fluoroscopic placement of a cryo-needle directly into the lesion. **C** Intraprocedural non-contrast CT scan obtained after 5 min during the second freezing cycle with good intraprocedural visualization of the ice ball. **D** Follow-up CT (portal venous phase) after 6 months showing local tumor control and intact adjacent intestinal structures. There were no complications

Published results indicate a strong consensus on the safety of percutaneous cryoablation in the liver. Various aspects of this therapeutic option have been studied extensively, in particular the application of cryoablation near critical structures. Several investigators conclude that cryoablation is a safe procedure for treating lesions adjacent to vessels, bile ducts, the gallbladder, liver capsule, intestines, adrenal gland, renal diaphragm, and abdominal wall [4, 20, 21, 24, 27]. Furthermore, some investigators focused on the use of cryoablation in treating small lesions. They concluded that it is feasible, particularly due to the better visibility of the ablation zone (Fig. 6) [19, 21, 25]. Another area of interest was the combination of cryoablation with TACE in treating large lesions, which has been shown to be effective, well-tolerated, and associated with better overall survival and a minimal complication rate, especially in cases of unresectable HCC [16, 32, 34]. Comparative studies have shown varying results in terms of safety profiles when comparing cryoablation with hyperthermic techniques like RFA and MWA. Most data indicate no significant safety difference [17, 30]. However, there are a few studies suggesting that cryoablation is safer in treating small periductal HCCs and significantly reduces local tumor progression compared to RFA [21]. Additionally, cryoablation is highlighted for its effectiveness and safety in liver cancers unsuitable for surgical resection, showing advantages over both RFA and MWA, and offers better local control of primary HCCs larger than 2 cm than RFA [18, 26]. Of course, in clinical practice, outcomes also depend on how much experience the interventionalist has with each technique.

While there is consensus about the safety of cryoablation, the understanding of cryoshock remains ambiguous. This is evident from our literature review: Yang et al. described symptoms in six patients, including chills, fever, tachycardia, tachypnea, and temporary renal damage [39]. Conversely, Littrup et al. reported cryoshock in three patients with rather atypical symptoms such as severe hemorrhage, which might suggest capsule cracking [23]. These discrepancies among reports likely arise from the lack of a definite definition for cryoshock. The most widely accepted description, provided by Hutchinson et al., characterizes cryoshock as a syndrome involving pleural effusion, thrombocytopenia, disseminated intravascular coagulation (DIC), acute renal failure, myoglobinemia, liver failure, acute respiratory distress syndrome, and hypotension, suggesting a conditions resembling sepsis but without an infectious cause. Unlike procedures that cauterize vessels within the ablation zone, cryoablation exposes the necrotic contents of the ablation zone to systemic circulation [40]. This exposure can trigger a systemic inflammatory response, leading to the complications just mentioned [41, 42]. Reviewing the literature, we noted that authors neither use a consistent terminology nor consistently differentiate between cryoschock and cryo-reaction or cryo-response, which is hypothesized to represent a less severe manifestation of the systemic inflammatory response that triggers cryoshock [41, 42]. This milder postablation syndrome encompasses an array of symptoms, which include low-grade fever, malaise, chills, nausea, and delayed pain. It typically occurs 48–72 h after the procedure and persists for up to 5 days [42]. If a cryo-reaction is suspected, it is crucial to rule out other causes that may mimic the symptoms, such as infections due to hospital-acquired pathogens or abscess formation as a complication of the procedure.

Our meta-analysis has several limitations. First, there was high inhomogeneity among the reported data, especially considering major complications (I^2^ = 82.87%), which is mainly attributable to the lack of using a standardized reporting system. While we classified reported complications retrospectively, future investigators should use a standardized classification system that ideally also considers the long-term outcomes of these complications [43]. Secondly, the inconsistent use of the term ‘cryoshock’ slightly distorts the pooled incidence. While our meta-analysis included every instance classified as cryoshock, some cases likely did not involve actual cryoshock (e.g., Littrup et al.). Therefore, the pooled proportion would likely be even lower than the 0.265% reported here. Third, we did not include case reports of cryoshock in our meta-analysis [44], given their lack of insight into the frequency of this complication within a cohort. Thus, on the other hand, our study may not encompass all published incidences of cryoshocks. Additionally, missing data might have impacted specific aspects of our analysis (e.g., the moderator analysis), potentially introducing further bias.

In conclusion, cryoablation for treating hepatic lesions is a safe procedure with a pooled proportion of major complications below 5%. With an incidence of less than 0.3%, potentially fatal cryoshock is rare and has not been reported in lesions smaller than 3 cm included in this study.
